# Construction and validation of a prognostic model for esophageal cancer based on prognostic-related RNA-binding protein

**DOI:** 10.1097/MD.0000000000039639

**Published:** 2024-09-13

**Authors:** Yinzhong Sha, Abdusemer Reyimu, Wen Liu, Chuanjiang He, Aihemaitijiang Kaisaier, Pawuziye Paerhati, Li Li, Xiaoguang Zou, Aimin Xu, Xiang Cheng, Maimaitituersun Abuduaini

**Affiliations:** a Department of Laboratory Medicine, The First People’s Hospital of Kashi, Kashi City, China; b The First People’s Hospital of Kashi, Kashi City, China.

**Keywords:** DKC1, esophageal carcinoma, prognostic, RBPs, risk model

## Abstract

**Background:**

Construction of a prognostic model for esophageal cancer (ESCA) based on prognostic RNA-binding proteins (RBPs) and preliminary evaluation of RBP function.

**Methods:**

RNA-seq data of ESCA was downloaded from The Cancer Genome Atlas database and mRNA was extracted to screen differentially expressed genes using R. After screening RBPs in differentially expressed genes, R packages clusterProfiler and pathview were used to analyze the RBPs for Gene Ontology enrichment and Kyoto Encyclopedia of Genes and Genomes pathway. Based on the prognosis-related RBPs, COX regression was used to establish the prognostic risk model of ESCA. Risk model predictive ability was assessed using calibration analysis, receiver operating characteristic curves, Kaplan–Meier curves, decision curve analysis, and Harrell consistency index (C-index). A nomogram was established by combining the risk model with clinicopathological features.

**Results:**

A total of 105 RBPs were screened from ESCA. A prognostic risk model consisting of 6 prognostic RBPs (ARHGEF28, BOLL, CIRBP, DKC1, SNRPB, and TRIT1) was constructed by COX regression analysis. The prognosis was worse in the high-risk group, and the receiver operating characteristic curve showed (area under the curve = 0.90) that the model better predicted patients’ 5-year survival. In addition, 6 prognostic RBPs had good diagnostic power for ESCA. In addition, a total of 39 mRNAs were identified as predicted target molecules for DKC1.

**Conclusion:**

ARHGEF28, BOLL, CIRBP, DKC1, SNRPB, and TRIT1, as RBPs, are associated with the prognosis of ESCA, which may provide new ideas for targeted therapy of ESCA.

## 1. Introduction

Esophageal cancer (ESCA), as the 7th most prevalent and 6th most lethal cancer worldwide, is one of the major threats to human health, and is relatively more harmful, especially among vulnerable populations.^[1]^ Data from the Global Burden of Disease study found that the number of deaths due to ESCA worldwide increased from 319,332 in 1990 to 498,067 in 2019, a relative increase of 55.97%.^[2]^ According to data from the Global Cancer Observation 2020 online platform (GLOBOCAN2020), the number of ESCA cases worldwide climbed to 604,100, accounting for 3.1% of all cancers, and 1 in 18 deaths due to cancer in 2020 were associated with ESCA.^[3]^ Currently, chemotherapy, radiotherapy, and surgery are the mainstay of treatment for ESCA, but disease progression and recurrence still occur in many treated patients. Molecular targeted therapy is a breakthrough and revolutionary development in the field of tumor treatment, which has a more “curative” effect than the 3 traditional treatments of chemotherapy, radiotherapy, and surgery. Therefore, in-depth study of the pathogenesis of ESCA and searching for related tumor markers are of great significance in identifying therapeutic targets and improving patients’ prognosis.

RNA-binding proteins (RBPs) are a class of proteins that can recognize and bind RNAs (both coding and non-coding RNAs) and function in conjunction with their binding regions.^[4]^ To date, 1542 RBPs genes have been screened and identified through the human genome.^[5]^ It has been pointed out that RBPs are key regulators in tumor progression and are involved in the whole process of malignant tumorigenesis and development, and their molecular mechanisms include participation in RNA selective splicing, polyadenylation, transcription, and translation regulation.^[6]^ Meanwhile, RBPs also form ribonucleoprotein complexes with intracellular proteins, coding, or non-coding RNAs and influence tumor progression.^[7]^ Despite numerous studies on RBPs, their functions have not been resolved, and in particular, the role of RBPs in ESCA is still rarely reported. Therefore, in this study, we collected ESCA expression profiles and clinical information from The Cancer Genome Atlas (TCGA) database, screened for differentially expressed RBPs in ESCA, and constructed a risk model based on prognosis-related RBPs by bioinformatics analysis, which can effectively differentiate the prognostic differences of ESCA patients and optimize the predictive effect of the current TNM staging system, with the aim of providing ESCA patients with a new model and a new idea for therapeutic decisions.

## 2. Materials and methods

### 2.1. Data acquisition and preprocessing

The ESCA-RNA-seq dataset was obtained from the TCGA database,^[8]^ including 160 ESCA samples and 11 normal samples. Ensembl IDs were converted to gene symbols using the gene annotation file (Homo_sapiens.GRCh38.99) (https://asia.ensembl.org/index.html). The edgeR package^[9]^ was used for quality control and preprocessing of TCGA data, creation of DGEList objects to integrate gene expression count matrices and sample information, and normalization of data using the TMM (trimmed mean of M values) method. |log2(fold change [FC])|>1 and *P* < .05 were used as inclusion criteria for differentially expressed genes (DEGs). 1542 RBPs were extracted from previous literature^[10]^ (Appendix 1, Supplemental Digital Content, http://links.lww.com/MD/N537), and differentially expressed RBPs were extracted from DEGs for further analysis.

### 2.2. GO functional annotation and KEGG enrichment analysis

The “clusterProfiler” and package of R software (Version 3.6.3) was used for Gene Ontology (GO) enrichment analysis and Kyoto Encyclopedia of Genes and Genomes (KEGG) signal pathway analysis of DEGs.^[11]^ The screening criteria were the differential genes on a single term, which was arranged according to the enrichment degree. *P* < .05 was considered as statistically significant. GO was used to analyze the functional enrichment in biological process (BP), cellular component (CC) and molecular function (MF).

### 2.3. Construction of prognostic risk model and its validity evaluation

Univariate Cox regression analysis was used to screen the genes associated with prognosis, and the indicators with *P* < .05 were selected for further analysis. Multivariate Cox regression analysis was used to construct the ESCA prognostic risk model. The risk score of each patient was calculated according to the model formula, and the patients were divided into high-risk and low-risk groups based on the median score. Heat maps of the expression trends of the 3 modeled genes were plotted using the “pheatmap” package in R software (version 3.6.3), and Kaplan–Meier (KM) survival analysis was used to compare the prognosis of the high-risk group with that of the low-risk group. Meanwhile, the predictive ability of the model was evaluated by plotting the receiver operating characteristic (ROC) curve and calculating the area under the curve. Univariate and multivariate Cox regression analyses were used to determine the correlation of risk values with clinical characteristics and overall survival (OS) in the TCGA cohort. A nomogram model of risk scores and clinicopathologic data related to prognosis was constructed using the “rms” package of R software (version 3.6.3). Nomogram was assessed by Harrell consistency index (C-index). Calibration curves, ROC curves were used to assess the accuracy of the nomogram in predicting 1-, 3-, and 5-year OS. In addition, decision curve analysis was used for clinical application prospect determination.

### 2.4. Expression and diagnostic analysis of model genes

Box plots were used to analyze the differences in expression of ARHGEF28, BOLL, CIRBP, DKC1, SNRPB, and TRIT1 in normal tissues versus ESCA, and ROC curves were used to examine the efficacy of the above genes in distinguishing ESCA from normal samples.

### 2.5. RBP target mRNAs exploration and analysis

StarBase online tool (http://starbase.sysu.edu.cn/) was used to predict the target mRNAs of the model RBPs. The model RBPs ARHGEF28, BOLL, CIRBP, SNRPB, and TRIT1 did not show a match. Therefore, we used DKC1 as a research target to predict its target mRNA. Therefore, DKC1 was used as a research target to predict its target mRNAs. The CLIP-seq types (PAR-CLIP, iCLIP, eCLIP, HITS-CLIP, Other CLIP) included in the database were utilized to predict the downstream mRNAs of DKC1. Screening conditions: pan-Cancer ≥ 1, CLIP region *P*-value≤.05. After obtaining RBP-mRNA interaction files, target mRNAs obtained by prediction were analyzed for differences (|logFC|>1, *P* < .05). The pearson method was used to analyze the expression correlation between differentially expressed target mRNAs and DKC1. The differentially expressed target mRNAs with significant correlation were imported into Cytoscape software for network visualization. The differentially expressed target mRNAs with significant correlation were analyzed for pathway enrichment, and the pathways with *P* < .05 were displayed in circle plots. Sankey diagram was used to show the DKC1–mRNA-pathway mechanism.

## 3. Results

### 3.1. Screening for differentially expressed RBPs in ESCA

**Figure 1.**
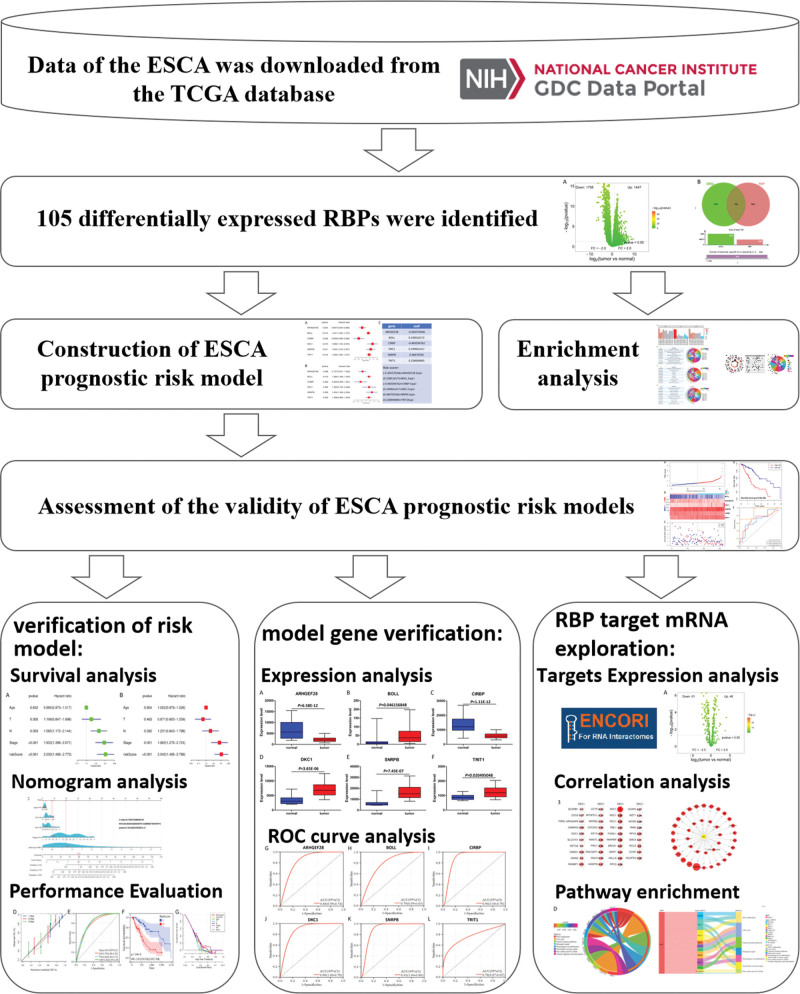
Research design and process of this study.

**Figure 2.**
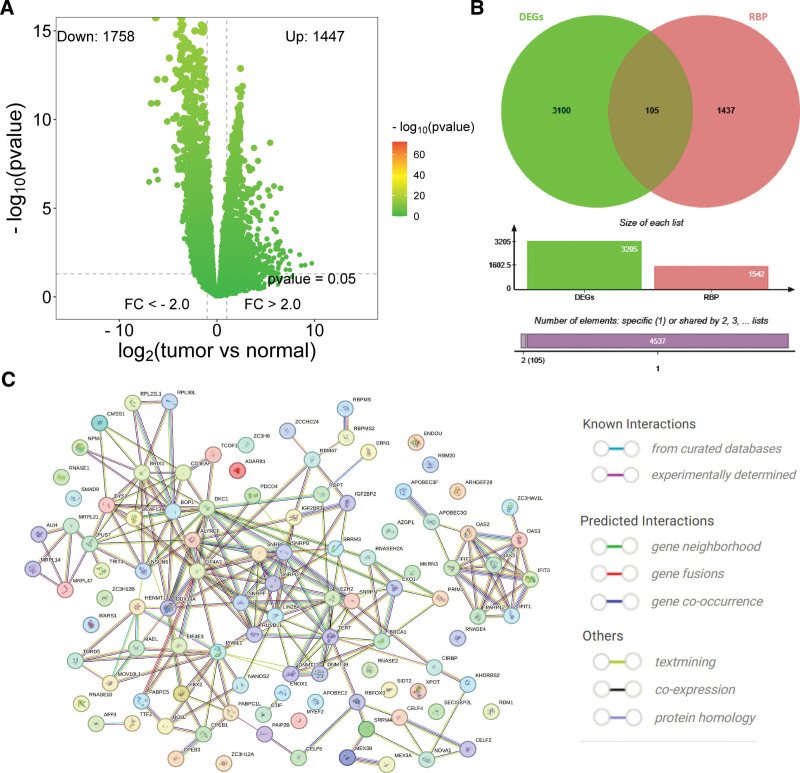
Screening of differentially expressed RBPs in ESCA. (A) Volcano map of DEGs screening between ESCA and normal tissues. The selection criteria were |logFC| > 1 and FDR value < 0.05. (B) Wayne diagrams were used to filter out RBPs from DEGs. (C) Interaction networks between differentially expressed RBPs.

The research route of this paper is shown in Figure 1. The gene expression profile data of ESCA and normal tissues in the TCGA database were analyzed using the edgeR package. A total of 3205 DEGs were screened in ESCA (Fig. 2A), which contained 105 RBPs (Fig. 2B). The 105 differentially expressed RBPs screened were imported into the STRING database to construct a protein–protein interaction network (Fig. 2C).

### 3.2. Enrichment analysis of differentially expressed RBPs

**Figure 3.**
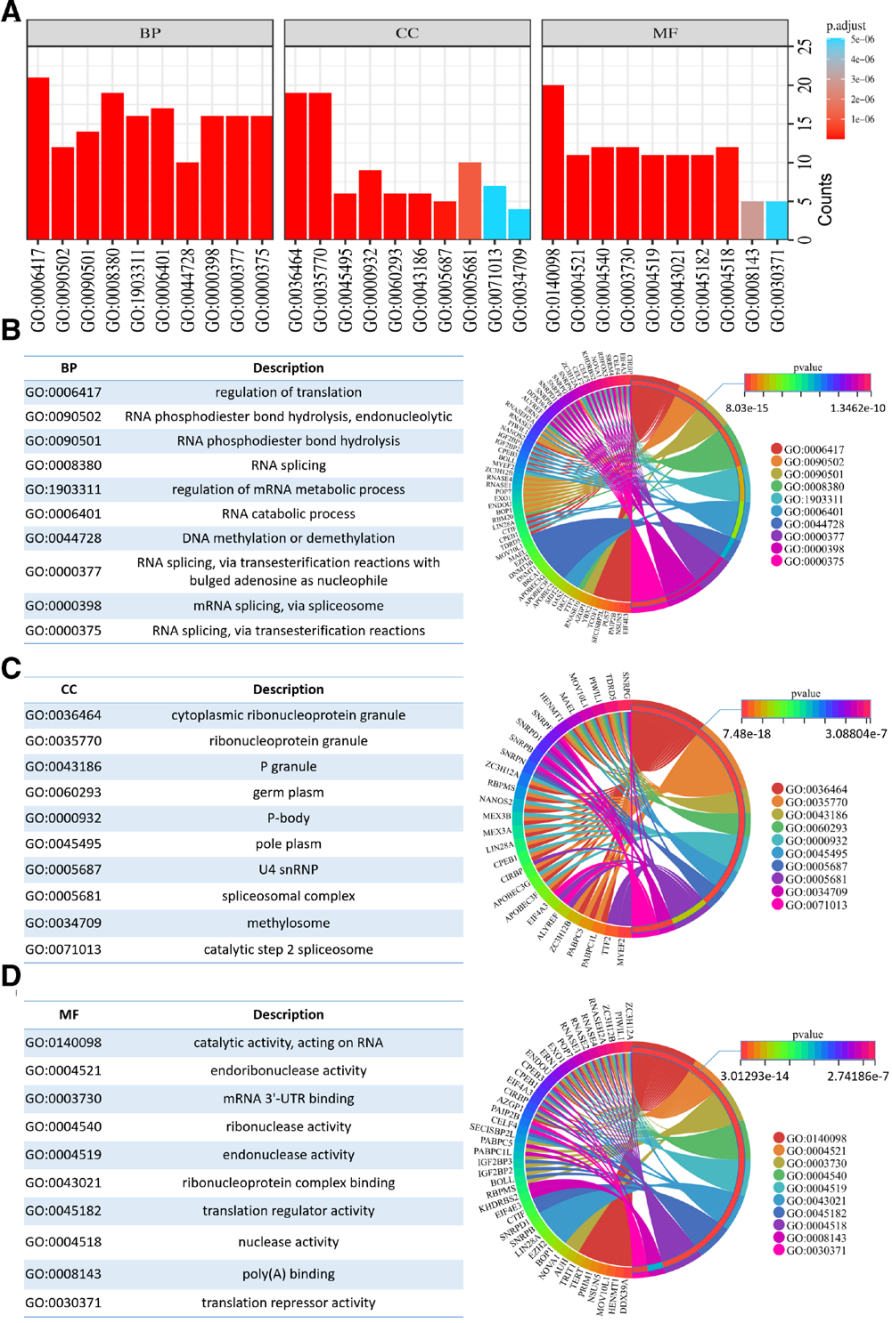
Functional enrichment analysis of 105 differentially expressed RBPs. (A) Top 10 terms significantly enriched in the BP, CC and MF groups, respectively. (B) Annotated items enriched in the BP group and the corresponding RBPs. (C) Annotated items enriched in the CC group and the corresponding RBPs. (D) Annotated items enriched in the MF group and the corresponding RBPs. (The *P*-value indicates the level of significance of the enriched entries.).

**Figure 4.**
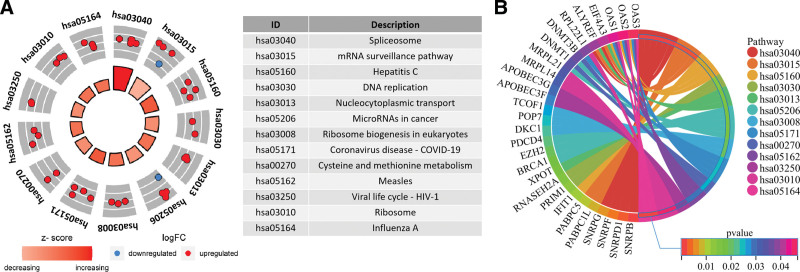
KEGG analysis of 105 differentially expressed RBPs. (A) Circle plots were used to show KEGG pathways in which RBPs were significantly enriched (The outer circle shows the scatter of RBPs corresponding to logFC in each term. Red dots indicate up-regulated RBPs and blue dots indicate down-regulated RBPs). (B) Distribution of RBPs in the KEGG pathway.

GO functional annotation and KEGG pathway enrichment analysis was performed on 105 differentially expressed RBPs using clusterProfiler and pathview packages. The significant GO analysis results were categorized into 3 groups, BP, CC, and MF, and the top 10 items in each group were taken (Fig. 3A). The BP group results showed that RBPs were mainly involved in regulation of translation, RNA phosphodiester bond hydrolysis endonucleolytic, RNA phosphodiester bond hydrolysis, RNA splicing, regulation of mRNA metabolic process, RNA catabolic process, DNA methylation or demethylation, RNA splicing via transesterification reactions with bulged adenosine as nucleophile, mRNA splicing via spliceosome, RNA splicing via transesterification reactions (Fig. 3B). The CC group results showed that RBPs were mainly involved in cytoplasmic ribonucleoprotein granule, ribonucleoprotein granule, P granule, germ plasm, P-body, pole plasm, U4 snRNP, the spliceosomal complex, methylosome, catalytic step 2 spliceosome (Fig. 3C). The MF group results showed that RBPs were mainly involved in catalytic activity, acting on RNA, endoribonuclease activity, mRNA 3’-UTR binding, ribonuclease activity, endonuclease activity, ribonucleoprotein complex binding, translation regulator activity, nuclease activity, poly(A) binding, translation repressor activity (Fig. 3D). KEGG pathway analysis showed that RBPs were mainly enriched in Spliceosome, mRNA surveillance pathway, hepatitis C, DNA replication, nucleocytoplasmic transport, microRNAs in cancer, and ribosome biogenesis in eukaryotes, Coronavirus disease – COVID-19, cysteine and methionine metabolism, measles, viral life cycle – HIV-1, ribosome, influenza A (Fig. 4A and B). All functional enrichment terms with significance were shown in Appendix 2, Supplemental Digital Content, http://links.lww.com/MD/N538.

### 3.3. Construction and validity assessment of risk models

**Figure 5.**
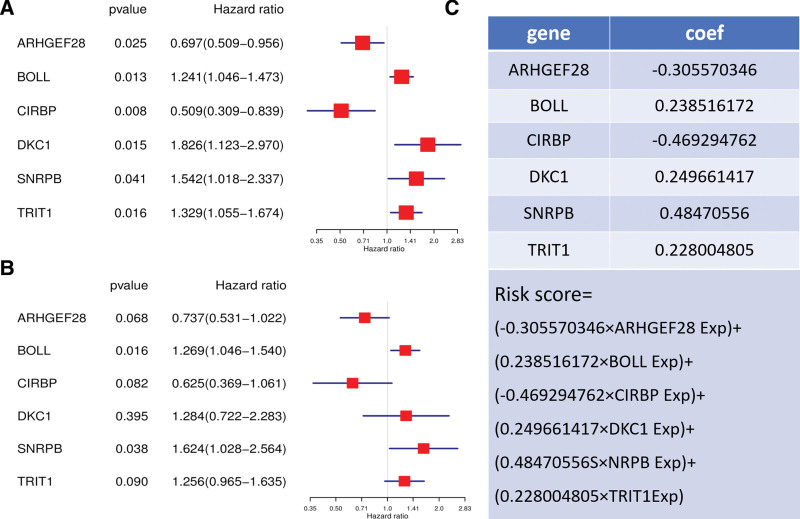
Risk modeling of ESCA patients based on prognostically relevant RBPs. (A) Screening of prognostically relevant RBPs by univariate COX survival analysis. (C) Multivariate COX survival analysis for independent prognostic analysis. (D) Risk score calculation formula based on 6 prognostically relevant RBPs.

**Figure 6.**
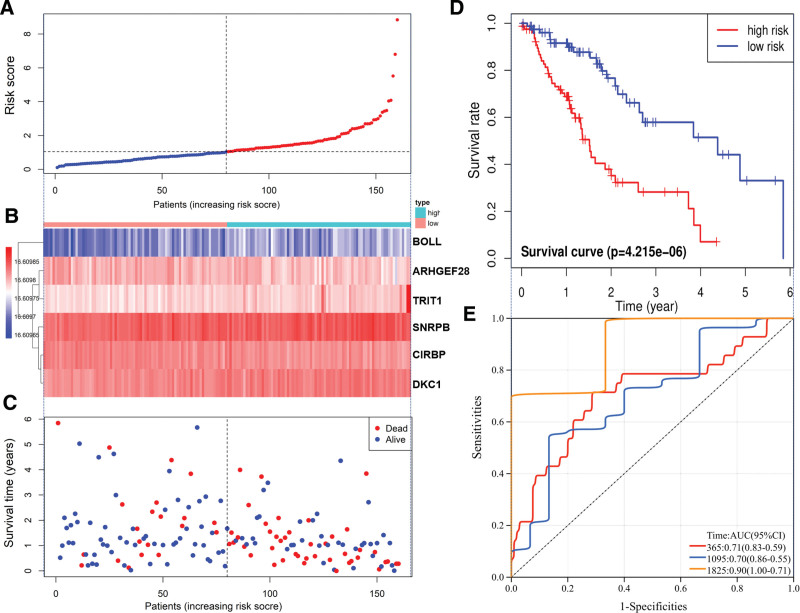
Performance evaluation of prognostic risk scoring model. (A) Classification of patients according to risk scores (red indicates high risk and blue indicates low risk). (B) Heatmap of expression trends of 3 model RBPs. (C) Distribution of survival status of patients in high-risk group and low-risk group (red indicates death and blue indicates survival). (D) KM survival analysis of patients in the high- and low-risk groups of ESCA. (E) ROC curves were used to analyze the predictive efficacy of risk scores on patient prognosis. AUC (Area Under Curve) indicates the area below the ROC curve. The value is between 0 and 1. The higher the value, the better the prediction effect of the model.

Univariate COX analysis of 105 RBPs showed that the expression of ARHGEF28, BOLL, CIRBP, DKC1, SNRPB, and TRIT1 was associated with ESCA prognosis (Fig. 5A). An ESCA prognostic risk model was constructed based on the RBPs associated with prognosis. Multivariate COX analysis showed that BOLL and SNRPB might be an independent prognostic factor for patients (Fig. 5B). The risk score in the model was calculated as follows: risk score = (−0.305570346 × ARHGEF28 Exp) + (0.238516172 × BOLL Exp) + (−0.469294762 × CIRBP Exp) + (0.249661417 × DKC1 Exp) + (0.48470556S × NRPB Exp) + (0.228004805 × TRIT1 Exp) (Fig. 5C). The risk scores of the patients were ranked and categorized into high-risk and low-risk groups (Fig. 6A). The heatmap showed the trend of the risk score versus the expression level of modeled RBPs (Fig. 6B). The shorter the survival time of the patients (Fig. 6C). The KM survival curve also confirmed that the survival rate of the high-risk group was significantly lower than that of the low-risk group (*P* = 4.215e−06) (Fig. 6D). The results of the ROC curve showed that the risk model had a good predictive ability for the 5-year survival rate of the cancer patients (Fig. 6E).

### 3.4. Clinical predictive value of risk models

**Figure 7.**
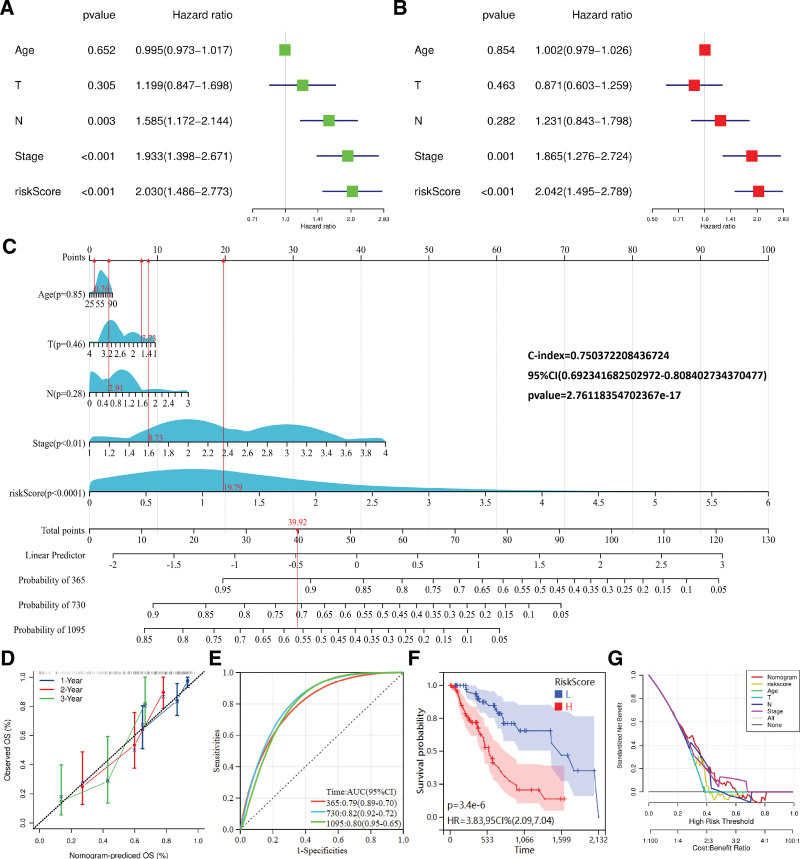
ESCA risk modeling based on prognostically relevant RBPs predicted patient prognosis (nomogram). (A) Univariate prognostic analysis of risk score and clinical characteristics. (B) Multivariate prognostic analysis of risk scores and clinical characteristics. (C) Nomogram of the prognostic model. (D) Calibration plot of the nomogram. (E) Time-dependent ROC analysis of nomogram predicting 1-, 2-, and 3-year OS in ESCA. (F) Efficacy of nomogram to predict prognosis in high and low risk patients. (G) Decision curve analysis of nomogram.

To further evaluate the predictive value of the above risk model, univariate and multivariate COX regression analyses were performed. Univariate COX analysis showed that N, Stage and risk score could be used as predictors of ESCA prognosis (Fig. 7A). Multivariate COX analysis showed that risk score could independently predict the prognosis of ESCA (HR = 2.042, *P* < .001) (Fig. 7B). The “rms” package was used to plot nomogram to predict survival in ESCA patients. Clinical factors and risk scores were used to predict survival in ESCA (Fig. 7C). The nomogram prediction model showed that risk score contributed the most to the prediction of prognosis (*P* < .0001). The nomogram diagram C-index value was 0.750372208436724 (*P* < .0001), indicating reliable performance of the nomogram. The calibration graph showed that the nomogram was suitable for predicting 1-, 2-, and 3-year OS in ESCA patients (Fig. 7D). The area under the curves for the prediction of 1-, 2-, and 3-year OS were 0.79, 0.82, and 0.80, respectively (Fig. 7E). KM curves showed a significant difference in survival between the high- and low-risk groups of ESCA patients in the nomogram (Fig. 7F). Decision curve analysis showed that nomogram performed better at threshold probability (ranging from 30–50%) (Fig. 7G).

### 3.5. Model RBPs expression and diagnostic analysis

**Figure 8.**
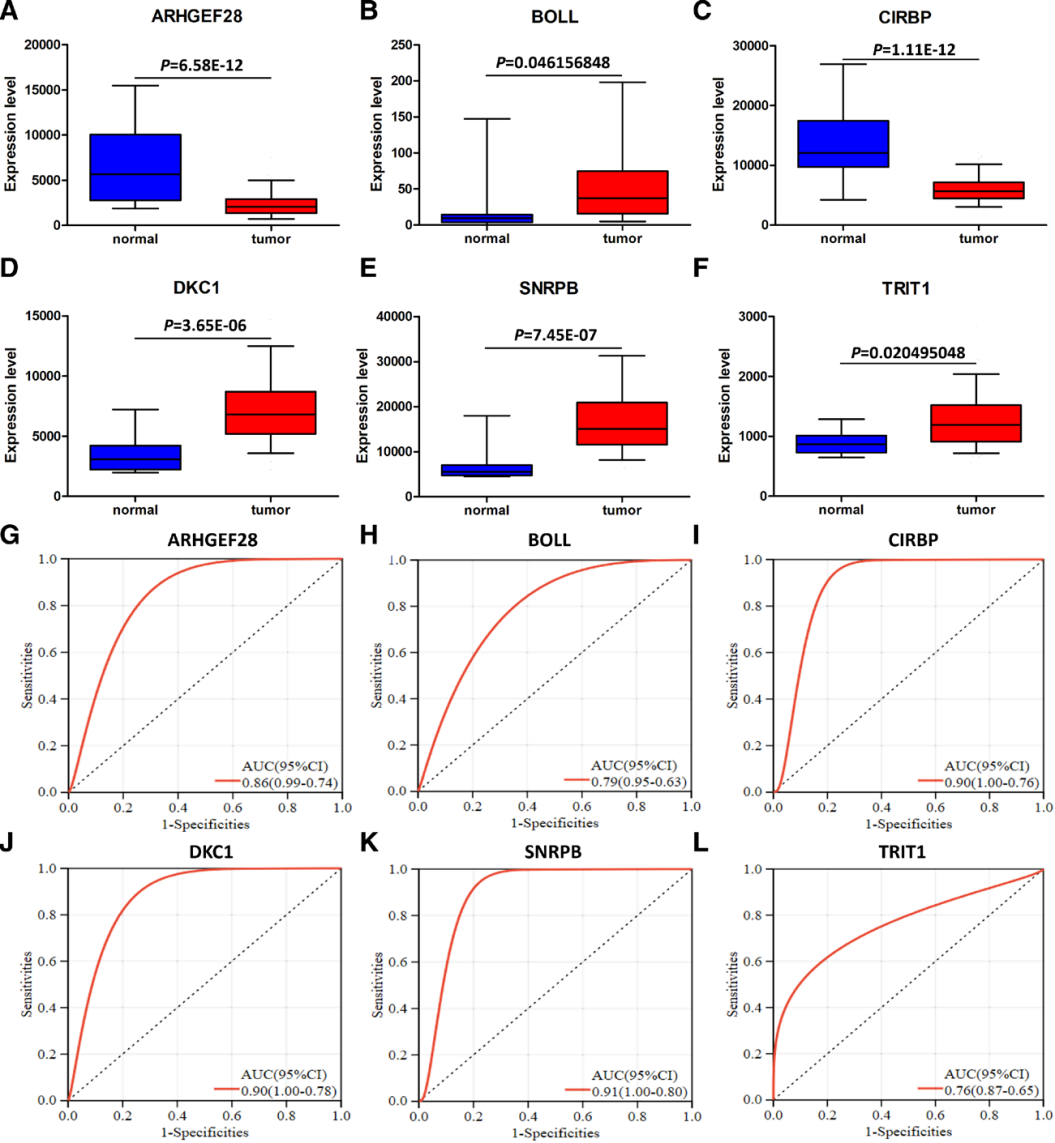
Model RBPs expression and diagnostic analysis. (A) Expression of ARHGEF28 in ESCA. (B) Expression of BOLL in ESCA. (C) Expression of CIRBP in ESCA. (D) Expression of DKC1 in ESCA. (E) Expression of SNRPB in ESCA. (F) Expression of TRIT1 in ESCA. (G) ROC curve was used to assess the diagnostic efficacy of ARHGEF28. (H) ROC curve was used to assess the diagnostic efficacy of BOLL. (I) ROC curve was used to assess the diagnostic efficacy of CIRBP. (J) ROC curve was used to assess the diagnostic efficacy of DKC1. (K) ROC curve was used to assess the diagnostic efficacy of SNRPB. (L) ROC curve was used to assess the diagnostic efficacy of TRIT1. *P < .05, **P < .01, ***P < .001.

Analysis of model RBPs revealed that the expression of BOLL, DKC1, SNRPB, and TRIT1 was up-regulated in ESCA tissues, and the expression of ARHGEF28 and CIRBP was down-regulated in ESCA tissues (Fig. 8A–F). All of the above model RBPs had high diagnostic potency for ESCA (Fig. 8G–L).

### 3.6. RBP target mRNAs exploration and analysis

**Figure 9.**
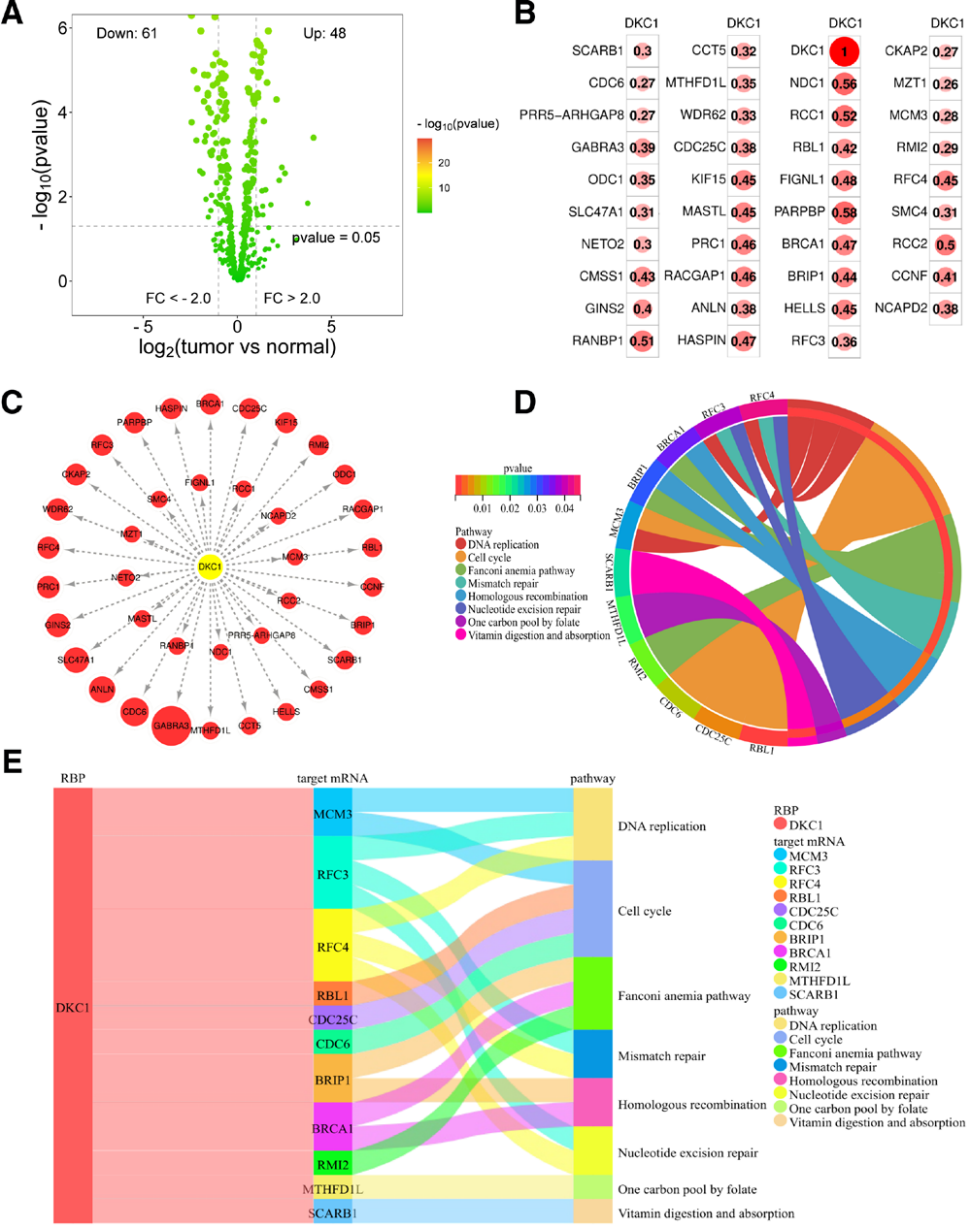
Exploration of RBP target mRNAs and pathway enrichment. (A) A total of 736 target mRNAs of DKC1 protein were predicted by StarBase online tool, of which 48 mRNAs were up-regulated and 61 mRNAs were down-regulated. (B) DKC1 was significantly correlated with the expression of 39 target mRNAs, and all were positively correlated (the numbers in the circles represent correlation coefficients). (C) The 39 differentially expressed target mRNAs with significant correlation with DKC1 were imported into Cytoscape software for network visualization (yellow nodes indicate RBP and red nodes indicate up-regulated target mRNAs) (the size of red nodes increases with the size of logFC of target mRNAs). (D) Pathway enrichment of 39 differentially expressed target mRNAs with significant correlation to DKC1 was analyzed. (E) Sankey diagram was used to show the DKC1–mRNAs-pathway mechanism.

ARHGEF28, BOLL, SNRPB, TRIT1, and CIRBP of RBPs in the StarBase database did not show matches. Therefore, target mRNAs for DKC1 were predicted. The prediction revealed that DKC1 may target 736 mRNAs. Among them, 109 mRNAs were differentially expressed in ESCA (Fig. 9A). Correlation analysis revealed that DKC1 was significantly correlated with the expression of 39 differentially expressed target mRNAs, and all of them were positively correlated (Fig. 9B). The 39 differentially expressed target mRNAs with significant correlation were imported into Cytoscape software for network visualization (Fig. 9C). KEGG pathway analysis revealed that the 39 differentially expressed target mRNAs with significant correlations were mainly enriched in DNA replication, cell cycle, Fanconi anemia pathway, mismatch repair, homologous recombination, nucleotide excision repair, one carbon pool by folate, vitamin digestion, and absorption (Fig. 9D). Finally, the DKC1–mRNAs-pathway mechanism was analyzed (Fig. 9E).

## 4. Discussion

RBPs are considered to be amplification factors driving oncogenic mutations. Numerous studies have demonstrated that RBPs play a crucial role in the development and progression of a variety of malignant tumors^[12]^ and are closely related to the prognosis of tumor patients. One study confirmed^[13]^ that pancreatic ductal adenocarcinoma patients with high ESRP1 expression have longer survival than those with its low expression. Although, previous studies have found that RBPs play an important role in ESCA,^[14]^ comprehensive studies on the function and prognosis of RBPs in ESCA have not been reported.

In this study, we systematically investigated the role and prognostic value of RBPs in ESCA. A total of 105 differentially expressed RBPs were identified between normal tissues and tumor tissues of ESCA using TCGA database. The potential functions of the screened RBPs were comprehensively analyzed by bioinformatics. Previous studies have found that aberrant regulation of translation, RNA processing and RNA metabolic process is the driving force behind the development of human diseases.^[15]^ Functional enrichment analysis revealed that differentially expressed RBPs in ESCA are mainly enriched in regulation of translation, RNA splicing, regulation of mRNA metabolic process, RNA catabolic process, DNA methylation or demethylation, and other BPs. methylation or demethylation and other BPs. Meanwhile, KEGG analysis revealed that the metabolic abnormalities of RBPs mainly acted in the process of RNA splicing and degradation, and the findings were consistent with those of previous studies.^[16]^ A total of 6 RBPs strongly associated with ESCA prognosis were screened by one-way COX analysis. A prognostic model was constructed based on the 6 prognostically relevant RBPs (ARHGEF28, BOLL, CIRBP, DKC1, SNRPB, and TRIT1) and validated by COX regression, which further validated the RBPs’ potential prognostic efficacy of RBPs in ESCA. Finally, the mechanism of action of DKC1 as an RBP was also explored.

Boule-like RNA-binding protein (BOLL) is a transcription factor and RNA-binding protein that is essential during mammalian testicular development and maturation, and is able to BOLL regulate the expression of various genes in the testis.^[17]^ For example, BOLL acts by binding to the 3’-UTR of mRNA and regulating its translation. In addition to its functions related to the reproductive system, other studies have shown that BOLL can also contribute to the development and progression of cancer.^[18]^ In this study, high expression of BOLL in ESCA was found to be associated with poor patient prognosis and was efficient in the diagnosis of ESCA. Enrichment analysis showed that BOLL was involved in regulation of translation, regulation of mRNA metabolic process, RNA catabolic process, mRNA 3’-UTR binding, translation regulator activity. It reveals that BOLL may be a potential therapeutic target for ESCA, while the targeting and regulatory mechanism of BOLL still needs further study.

Dyskeratosis congenita 1 (DKC1) is a human X-linked gene encoding the ribonucleoprotein dyskeratosis protein (Dyskerin). The gene is 16kb in length, consists of a total of 15 exons, and is localized to chromosome Xq28. DKC1 is an important component of the telomerase complex, which plays an important role in the maintenance of normal telomere function, precursor rRNA processing, post-transcriptional modification of genes and normal ribosome biosynthesis.^[19]^ Some studies have shown that the expression of DKC1 is significantly up-regulated in diffuse large B lymphoblastoma and breast cancer,^[20]^ and DKC1 can affect the proliferation, invasion and metastasis of a variety of tumors, such as colon cancer, glioma, renal cancer, etc.^[21–23]^ In this study, high expression of DKC1 in ESCA was found to correlate with poor patient prognosis and was highly efficient in the diagnosis of ESCA. Enrichment analysis revealed that DKC1 was involved in the biological function of RNA catabolic process and in Ribosome biogenesis in eukaryotes pathway. It has been shown that there is a relationship between Dyskerin protein and RNA catabolic process. Specifically, Dyskerin proteins promote the stability of certain mRNAs and maintain their stability in cells by inhibiting their degradation process. For example, in humans, Dyskerin binds to the 3’ UTR region of mRNAs, which enables it to inhibit the rate of their degradation by RNA endonucleases (RNases), thereby regulating gene expression and RNA metabolism. In this study, the target mRNAs of DKC1 were predicted, and a total of 39 target mRNAs were found to be differentially expressed in ESCA and significantly correlated with the expression of DKC1. In addition, 39 mRNAs were pathway-enriched and found to be associated with important pathways such as DNA replication and cell cycle. The above results revealed the DKC1–mRNAs-pathway mechanism and provided a theoretical basis for the targeted therapy of ESCA.

Small nuclear ribonucleoprotein-associated proteins B (SNRPB), as a core component of the SMN-Sm complex that mediates the assembly of the spliceosome snRNP, as well as a component of the building blocks of the spliceosome, U1, U2, U4, and U5 small nuclear ribonucleoproteins (snRNPs), play a role in pre-mRNA splicing.^[24,25]^ Studies have confirmed that inhibition of SNRP gene expression will lead to disruption of its regulation of downstream genes making cancer cells less viable.^[25]^ SNRPB as a SNRP protein can promote the progression of a variety of cancers. For example, SNRPB is able to promote hepatocellular carcinoma proliferation^[26]^ as well as glioma cell growth.^[27]^ In this study, high expression of SNRPB in ESCA was found to be associated with poor patient prognosis and was efficient in the diagnosis of ESCA. Enrichment analysis showed that SNRPB was involved in RNA splicing, mRNA splicing, U4 snRNP, spliceosomal complex, methylosome, catalytic step 2 spliceosome, ribonucleoprotein complex binding, Spliceosome pathway. It reveals that SNRPB may be a potential therapeutic target for ESCA, and the target-regulatory mechanism of SNRPB still needs further study.

This study has several potential limitations. First, this study relied on a prognostic model constructed from RNA-seq data of ESCA patients in the TCGA database, but external validation is still needed. Second, the relatively small sample size may bias the results of the study. To further confirm the reliability of our prognostic model, we plan to externally validate the model in future studies and seek a larger sample set to strengthen our conclusions.

## 5. Conclusion

In conclusion, the 6 prognostically relevant RBPs mentioned above (ARHGEF28, BOLL, SNRPB, TRIT1, and CIRBP) were used to construct the ESCA risk model. The distribution of survival status indicated that the death population was more intensive in the high-risk group, and the prognosis of patients in the high-risk subgroup was poorer. The model had a high predictive efficiency for 5-year survival of ESCA patients. Multivariate COX analysis and nomogram showed that model scores independently predicted the prognosis of ESCA. There are potentially 39 regulatory target mRNAs of DKC1 as model RBPs, and the target mRNAs act in important pathways such as DNA replication, cell cycle, and so on. It reveals that DKC1 may be a potential therapeutic target for ESCA.

## Acknowledgments

We would like to thank the team members for their contributions to this paper, and then we will continue to work hard to do relevant research.

## Author contributions

**Data curation:** Abdusemer Reyimu, Wen Liu, Chuanjiang He, Aihemaitijiang Kaisaier, Pawuziye Paerhati.

**Formal analysis:** Abdusemer Reyimu, Wen Liu, Aihemaitijiang Kaisaier, Pawuziye Paerhati.

**Methodology:** Abdusemer Reyimu, Wen Liu, Chuanjiang He.

**Project administration:** Yinzhong Sha, Li Li, Xiaoguang Zou, Aimin Xu, Xiang Cheng, Maimaitituersun Abuduaini.

**Writing – original draft:** Yinzhong Sha, Abdusemer Reyimu.

**Writing – review & editing:** Li Li, Xiaoguang Zou, Aimin Xu, Xiang Cheng, Maimaitituersun Abuduaini.

## Supplementary Material



## References

[1] Rogers JE Sewastjanow-Silva M Waters RE Ajani JA. Esophageal cancer: emerging therapeutics. Expert Opin Ther Targets. 2022;26:107–17. 35119973 10.1080/14728222.2022.2036718

[2] GBD 2019 Diseases and Injuries Collaborators. Global burden of 369 diseases and injuries in 204 countries and territories, 1990-2019: a systematic analysis for the Global Burden of Disease Study 2019. Lancet. 2020;396:1204–22. 33069326 10.1016/S0140-6736(20)30925-9 PMC7567026

[3] Sung H Ferlay J Siegel RL. Global cancer statistics 2020: GLOBOCAN estimates of incidence and mortality worldwide for 36 cancers in 185 countries. CA Cancer J Clin. 2021;71:209–49. 33538338 10.3322/caac.21660

[4] Hong S. RNA binding protein as an emerging therapeutic target for cancer prevention and treatment. J Cancer Prev. 2017;22:203–10. 29302577 10.15430/JCP.2017.22.4.203 PMC5751837

[5] Gerstberger S Hafner M Tuschl T. A census of human RNA-binding proteins. Nat Rev Genet. 2014;15:829–45. 25365966 10.1038/nrg3813 PMC11148870

[6] Pereira B Billaud M Almeida R. RNA-binding proteins in cancer: old players and new actors. Trends Cancer. 2017;3:506–28. 28718405 10.1016/j.trecan.2017.05.003

[7] Liang G Meng W Huang X. miR-196b-5p-mediated downregulation of TSPAN12 and GATA6 promotes tumor progression in non-small cell lung cancer. Proc Natl Acad Sci U S A. 2020;117:4347–57. 32041891 10.1073/pnas.1917531117 PMC7049122

[8] Wei L Jin Z Yang S Xu Y Zhu Y Ji Y. TCGA-assembler 2: software pipeline for retrieval and processing of TCGA/CPTAC data. Bioinformatics. 2018;34:1615–7. 29272348 10.1093/bioinformatics/btx812 PMC5925773

[9] Robinson MD McCarthy DJ Smyth GK. edgeR: a Bioconductor package for differential expression analysis of digital gene expression data. Bioinformatics. 2010;26:139–40. 19910308 10.1093/bioinformatics/btp616 PMC2796818

[10] Merkley MA Hildebrandt E Podolsky RH. Large-scale analysis of protein expression changes in human keratinocytes immortalized by human papilloma virus type 16 E6 and E7 oncogenes. Proteome Sci. 2009;7:29. 19698150 10.1186/1477-5956-7-29 PMC2744660

[11] Wu T Hu E Xu S. clusterProfiler 4.0: a universal enrichment tool for interpreting omics data. Innovation (Camb). 2021;2:100141. 34557778 10.1016/j.xinn.2021.100141 PMC8454663

[12] Yu D Zhang C Gui J. RNA-binding protein HuR promotes bladder cancer progression by competitively binding to the long noncoding HOTAIR with miR-1. Onco Targets Ther. 2017;10:2609–19. 28553126 10.2147/OTT.S132728 PMC5440069

[13] Ueda J Matsuda Y Yamahatsu K. Epithelial splicing regulatory protein 1 is a favorable prognostic factor in pancreatic cancer that attenuates pancreatic metastases. Oncogene. 2014;33:4485–95. 24077287 10.1038/onc.2013.392 PMC4041859

[14] Reyimu A Xing F Zhou W. Screening of potential key genes in esophageal cancer based on RBP and expression verification of HENMT1. Medicine (Baltim). 2023;102:e36544. 10.1097/MD.0000000000036544 PMC10713111 38065897

[15] Siang D Lim YC Kyaw A. The RNA-binding protein HuR is a negative regulator in adipogenesis. Nat Commun. 2020;11:213. 31924774 10.1038/s41467-019-14001-8 PMC6954112

[16] Li W Gao LN Song PP You C-G. Development and validation of a RNA binding protein-associated prognostic model for lung adenocarcinoma. Aging (Albany NY). 2020;12:3558–73. 32087603 10.18632/aging.102828 PMC7066909

[17] Li T Wang X Zhang H Chen Z Zhao X Ma Y. Histomorphological comparisons and expression patterns of BOLL gene in sheep testes at different development stages. Animals (Basel). 2019;9:105. 30901845 10.3390/ani9030105 PMC6466207

[18] Kang KJ Pyo JH Ryu KJ. Oncogenic role of BOLL in colorectal cancer. Dig Dis Sci. 2015;60:1663–73. 25605553 10.1007/s10620-015-3533-z

[19] Mochizuki Y He J Kulkarni S Bessler M Mason PJ. Mouse dyskerin mutations affect accumulation of telomerase RNA and small nucleolar RNA, telomerase activity, and ribosomal RNA processing. Proc Natl Acad Sci U S A. 2004;101:10756–61. 15240872 10.1073/pnas.0402560101 PMC490007

[20] Poncet D Belleville A T’Kint DRC. Changes in the expression of telomere maintenance genes suggest global telomere dysfunction in B-chronic lymphocytic leukemia. Blood. 2008;111:2388–91. 18077792 10.1182/blood-2007-09-111245

[21] Hou P Shi P Jiang T. DKC1 enhances angiogenesis by promoting HIF-1alpha transcription and facilitates metastasis in colorectal cancer. Br J Cancer. 2020;122:668–79. 31857720 10.1038/s41416-019-0695-z PMC7054532

[22] Zhang M Pan Y Jiang R. DKC1 serves as a potential prognostic biomarker for human clear cell renal cell carcinoma and promotes its proliferation, migration and invasion via the NF-kappaB pathway. Oncol Rep. 2018;40:968–78. 29901172 10.3892/or.2018.6484

[23] Miao FA Chu K Chen HR. Increased DKC1 expression in glioma and its significance in tumor cell proliferation, migration and invasion. Invest New Drugs. 2019;37:1177–86. 30847721 10.1007/s10637-019-00748-w

[24] Liu N Chen A Feng N Liu X Zhang L. SNRPB is a mediator for cellular response to cisplatin in non-small-cell lung cancer. Med Oncol. 2021;38:57. 33835288 10.1007/s12032-021-01502-0

[25] Liu N Wu Z Chen A. SNRPB promotes the tumorigenic potential of NSCLC in part by regulating RAB26. Cell Death Dis. 2019;10:667. 31511502 10.1038/s41419-019-1929-y PMC6739327

[26] Zhan YT Li L Zeng TT Zhou N-N Guan X-Y Li Y. SNRPB-mediated RNA splicing drives tumor cell proliferation and stemness in hepatocellular carcinoma. Aging (Albany NY). 2020;13:537–54. 33289700 10.18632/aging.202164 PMC7834993

[27] Correa BR de Araujo PR Qiao M. Functional genomics analyses of RNA-binding proteins reveal the splicing regulator SNRPB as an oncogenic candidate in glioblastoma. Genome Biol. 2016;17:125. 27287018 10.1186/s13059-016-0990-4 PMC4901439

